# *AKR1A1* Variant Associated With Schizophrenia Causes Exon Skipping, Leading to Loss of Enzymatic Activity

**DOI:** 10.3389/fgene.2021.762999

**Published:** 2021-12-06

**Authors:** Kyoka Iino, Kazuya Toriumi, Riko Agarie, Mitsuhiro Miyashita, Kazuhiro Suzuki, Yasue Horiuchi, Kazuhiro Niizato, Kenichi Oshima, Atsushi Imai, Yukihiro Nagase, Itaru Kushima, Shinsuke Koike, Tempei Ikegame, Seiichiro Jinde, Eiichiro Nagata, Shinsuke Washizuka, Toshio Miyata, Shunya Takizawa, Ryota Hashimoto, Kiyoto Kasai, Norio Ozaki, Masanari Itokawa, Makoto Arai

**Affiliations:** ^1^ Schizophrenia Research Project, Department of Psychiatry and Behavioral Sciences, Tokyo Metropolitan Institute of Medical Science, Tokyo, Japan; ^2^ Department of Psychiatry, Tokyo Metropolitan Matsuzawa Hospital, Tokyo, Japan; ^3^ Department of Psychiatry, Takatsuki Hospital, Hachioji, Tokyo, Japan; ^4^ Department of Psychiatry, Graduate School of Medicine, Shinshu University, Nagano, Japan; ^5^ Department of Psychiatry, Nagoya University Graduate School of Medicine, Nagoya, Japan; ^6^ Medical Genomics Center, Nagoya University Hospital, Nagoya, Japan; ^7^ Department of Neuropsychiatry, Graduate School of Medicine, The University of Tokyo, Tokyo, Japan; ^8^ Department of Neurology, Tokai University School of Medicine, Isehara, Japan; ^9^ Division of Molecular Medicine and Therapy, Tohoku University Graduate School of Medicine, Sendai, Japan; ^10^ Department of Pathology of Mental Diseases, National Institute of Mental Health, National Center of Neurology and Psychiatry, Kodaira, Japan; ^11^ The International Research Center for Neurointelligence (WPI-IRCN) at The University of Tokyo Institutes for Advanced Study (UTIAS), Aoba-ku, Sendai, Japan

**Keywords:** aldo-keto reductase family 1 member A1, treatment-resistant schizophrenia, single nucleotide variant (SNV), exon skipping, frameshift mutation, glucuronate

## Abstract

Schizophrenia is a heterogeneous psychiatric disorder characterized by positive symptoms such as hallucinations and delusions, negative symptoms such as anhedonia and flat affect, and cognitive impairment. Recently, glucuronate (GlucA) levels were reported to be significantly higher in serum of patients with schizophrenia than those in healthy controls. The accumulation of GlucA is known to be related to treatment-resistant schizophrenia, since GlucA is known to promote drug excretion by forming conjugates with drugs. However, the cause of GlucA accumulation remains unclear. Aldo-keto reductase family one member A1 (AKR1A1) is an oxidoreductase that catalyzes the reduction of GlucA. Genetic loss of AKR1A1 function is known to result in the accumulation of GlucA in rodents. Here, we aimed to explore genetic defects in *AKR1A1* in patients with schizophrenia, which may result in the accumulation of GlucA. We identified 28 variants of *AKR1A1* in patients with schizophrenia and control subjects. In particular, we identified a silent c.753G > A (rs745484618, p. Arg251Arg) variant located at the first position of exon 8 to be associated with schizophrenia. Using a minigene assay, we found that the c.753G > A variant induced exon 8 skipping in AKR1A1, resulting in a frameshift mutation, which in turn led to truncation of the AKR1A1 protein. Using the recombinant protein, we demonstrated that the truncated AKR1A1 completely lost its activity. Furthermore, we showed that *AKR1A1* mRNA expression in the whole blood cells of individuals with the c.753G > A variant tended to be lower than that in those without the variants, leading to lower AKR activity. Our findings suggest that *AKR1A1* carrying the c.753G > A variant induces exon skipping, leading to a loss of gene expression and enzymatic activity. Thus, GlucA patients with schizophrenia with the c.753G > A variant may show higher GlucA levels, leading to drug-resistant schizophrenia, since drug excretion by GlucA is enhanced.

## Introduction

Schizophrenia is a complex and heterogeneous psychiatric disorder caused by genetic and environmental factors with a worldwide prevalence of approximately 1% (Owen et al., 2016). Recently, it has been reported that unmedicated patients with schizophrenia show increased levels of glucuronate (GlucA) in the peripheral blood compared with those in healthy controls, which can be improved by treatment with risperidone (Xuan et al., 2011). These findings suggest that GlucA in the blood might be useful as a metabolic biomarker for schizophrenia. Moreover, the accumulation of GlucA might be related to drug-resistant schizophrenia, since GlucA is known to promote drug excretion by forming conjugates with drugs (Mazerska et al., 2016). However, little is known about the molecular mechanisms underlying GlucA accumulation in patients with schizophrenia.

Aldo-keto reductase family one member A1 (AKR1A1) is an approximately 40 kDa monomeric oxidoreductase that is ubiquitously expressed throughout the body, especially in the kidneys and liver. AKR1A1 displays a broad spectrum of substrate activity and detoxifies aldehyde and carbonyl compounds such as methylglyoxal and 3-deoxyglucosone in a nicotinamide adenine dinucleotide phosphate (NADPH)-dependent manner (Petrash and Srivastava, 1982; Bohren et al., 1989; Spite et al., 2007; Kurahashi et al., 2014; Fujii et al., 2021). Furthermore, AKR1A1 catalyzes the reduction of GlucA in the biosynthesis of ascorbic acid in rodents (Gabbay et al., 2010; Takahashi et al., 2012). Inhibition of AKR1A1 in mice increases urinary output of GlucA (Barski et al., 2005). These findings suggest that AKR1A1 dysfunction leads accumulation of GlucA.

In the present study, we aimed to analyze genetic defects in *AKR1A1* in patients with schizophrenia and identify the molecular mechanisms that cause the accumulation of GlucA.

## Materials and Methods

### Human Subjects

Blood samples were obtained from 808 patients with schizophrenia (mean age: 49.0 years [standard deviation [SD]: 14.2 years]) and 636 healthy control subjects (mean age: 40.6 years [SD: 13.0 years]) for genome resequencing of *AKR1A1*. Patients were randomly recruited inpatients and outpatients. The cases included 437 men (mean age: 48.5 years [SD: 14.0 years]) and 417 women (mean age: 49.5 years [SD: 14.5 years]). Control subjects comprised 283 men (mean age: 41.5 years [SD: 13.6 years]) and 424 women (mean age: 40.1 years [SD: 12.5 years]). We did not assess the association between common variants and schizophrenia as the aim of this study was to focus on rare variations to reveal large biological effects, thus enabling clarification of pathophysiology in rare cases of schizophrenia. Therefore, these samples were not matched for age or sex. Schizophrenia was diagnosed according to the Diagnostic and Statistical Manual of Mental Disorders, fourth edition (4th ed., text rev.; DSM–IV–TR; American Psychiatric Association, 2000) to obtain a best-estimate lifetime psychiatric diagnosis, with consensus from at least two experienced psychiatrists. No structured interviews were conducted. The available medical records and family informant reports were also taken into consideration. Peripheral blood cells were obtained from patients with schizophrenia with/without the c.753G > A variant (*n* = 6) and two healthy controls among the subjects included in the genetic study. All participants provided written informed consent, and the study protocols were approved by the ethics committees of the Tokyo Metropolitan Institute of Medical Science (approval no. 20-17) and Tokyo Metropolitan Matsuzawa Hospital (approval no. 2018-8).

### Sequencing

Genome was purified from whole blood by SRL (Tokyo, Japan), which is a private clinical laboratory test company. Polymerase chain reaction (PCR) amplification was performed using the primer sets and PCR kits (Supplementary Table S1) as per the manufacturer’s instructions. All coding regions, exon–intron boundaries, 5′ untranslated region (UTR), and 3′ UTR of *AKR1A1* were examined by directly sequencing the PCR products using BigDye Terminator Cycle Sequencing Kit (Applied Biosystems, Foster City, CA, United States) and ABI PRISM 3130/3500 Genetic Analyzer (Applied Biosystems). We read both strands when an inserted or deleted nucleotide yielded dual signals derived from the wild-type (WT) and mutant strands. *AKR1A1* sequence analysis was performed based on RefSeq NM_006066. Variant data we found are deposited in the NCBI dbSNP: NCBI_subsnp# are 2137544288 and 5316170415–5316170441.

### Minigene Constructs

The exons 7–9 and the internal introns in *AKR1A1* gene were amplified from the human genome using the *AKR1A1*_ex7-9_fw and *AKR1A1*_ex7-9_rv primers (Supplementary Table S2) and inserted into the pTA2 cloning vector. Then, the *AKR1A1* minigene in the pTA2 vector was cut using HindIII and PstI enzymes and transferred into the pAcGFP1-C1 vector at the same site.

A c.753G > A mutant at the first position of exon 8 was produced using the KOD-plus- Mutagenesis Kit (Toyobo, Tokyo, Japan). For mutagenesis, *AKR1A1*_ex7-9_c.753_fw and *AKR1A1*_ex7-9_c.753_rv primers were used (Supplementary Table S2).

### Cell Culture and Transfection

HEK293, SH-SY5Y, and 1321N1 cells were maintained in Dulbecco’s modified Eagle’s medium (Gibco, Waltham, MA, United States) with 10% fetal bovine serum (Life Technologies, Carlsbad, CA, United States), 100 U/ml penicillin, and 100 μg/ml streptomycin (Gibco). At ∼80% confluency in a 10 cm dish, 5 μg of DNA constructs were transfected using FuGENE6 transfection reagent (Promega, Madison, WI, United States) according to the manufacturer’s protocol. The cells were collected 48 h after transfection.

### RNA Extraction and Reverse Transcription-PCR

Total RNA was extracted from HEK293, SH-SY5Y, and 1321N1 cells using the SV Total RNA Isolation System (Promega) and the extracted RNA was purified using the RNeasy MinElute Cleanup Kit (QIAGEN, Hilden, Germany) according to the manufacturer’s protocol. The obtained RNA was quantified using NanoDrop spectrophotometer (Thermo Fisher Scientific, Waltham, MA, United States), and 500 ng of the purified RNA was used as a template for complementary DNA (cDNA) synthesis using ReverTra Ace qPCR RT Kit (Toyobo). To investigate exon skipping, 10% of the synthesized cDNA was amplified using the pAcGFP1-C1_fw and AKex9_PstI_rv primers (Supplementary Table S2).

### Cloning and Mutagenesis

Total RNA derived from a human postmortem brain tissues was prepared using the SV Total RNA Isolation System (Promega), cleaned with an RNeasy MinElute Cleanup Kit (QIAGEN, Hilden, Germany) according to the manufacturer’s protocol. A High-Capacity cDNA Archive Kit (Applied Biosystems, Foster City, California) was used for reverse transcription of total RNA to first-strand cDNA synthesis according to the supplier’s protocols.

Full-length *AKR1A1* was cloned from the cDNA library using h*AKR1A1*_fw and h*AKR1A1*_rv primers (Supplementary Table S2). The amplified products were inserted into the pTA2 vector using TArget Clone (Toyobo). *AKR1A1* mutants, c.753G > A and c.264delC, were produced using pTA2_*AKR1A1* as a template with the KOD-plus-Mutagenesis Kit (Toyobo). *AKR1A1*_c.753_fw1 and rv1 primers were used for the c.753G > A mutant, and *AKR1A1*_c.264_fw1 and rv1 primers were used for the c.264delC mutant (Supplementary Table S2). Moreover, to delete the extra sequence below the new termination codon caused by the frameshift mutation, the c.753G > A and c.264delC mutants were amplified using the primer sets *AKR1A1*_c.753/c.264_fw2 and *AKR1A1*_c.753_rv2 and *AKR1A1*_c.264_rv2, respectively, and then self-ligated.

### Construction of the Recombinant Plasmid and Purification of the GST Fusion Protein

pTA2_*AKR1A1*_WT, pTA2_*AKR1A1*_c.753, and pTA2_*AKR1A1*_c.264 were used as templates for amplification of *AKR1A1* with EcoRI and SalI restriction enzyme cut sites at the 5′ and 3′ termini, respectively. The EcoRI_*AKR1A1*_fw and *AKR1A1*_SalI_WT_rv primers for the amplification of *AKR1A1* WT, *AKR1A1*_SalI_c.753_rv primer for the amplification of *AKR1A1* c.753, and *AKR1A1*_SalI_c.264_rv primer were employed for the amplification of *AKR1A1* c.264 (Supplementary Table S2). After amplification of *AKR1A1* with EcoRI and SalI restriction enzyme cut sites, the products were cut with EcoRI and SalI enzymes. The pGEX-4T-2 plasmid was cut with EcoRI and XhoI enzymes and ligated together to make the plasmid pGEX-4T-2_*AKR1A1*. The sequence of pGEX-4T-2_*AKR1A1* was confirmed using the pGEX_fw and pGEX_rv primers (Supplementary Table S2).

BL21 *Escherichia coli* transformed with pGEX-4T-2_*AKR1A1* was added to 3 ml of Luria Bertani medium containing 100 μg/ml ampicillin and incubated at 37°C with shaking. After 12–24 h, the optical density at 600 nm (OD_600_) was measured with GloMax Explorer Multimode Microplate Reader (Promega). The culture was diluted to OD = 0.4–0.5 with Luria Bertani medium containing 100 μg/ml ampicillin and incubated at 37°C with shaking. After 1 h, the culture was induced with 30 µl of 100 mM isopropyl *β*-D-1-thiogalactopyranoside and incubated at 37°C with shaking for 3 h. Cells were harvested by centrifugation at 20,400 × *g* for 1 min.

Glutathione-S-transferase (GST) fusion AKR1A1 from BL21 *E. coli* transformed with pGEX-4T-2_AKR1A1 was purified using the MagneGST Protein Purification System (Promega) according to the manufacturer’s protocols. Protein concentrations were determined using the bicinchoninic acid protein assay with GloMax Explorer Multimode Microplate Reader (Promega). Finally, we verified the purification of GST-AKR1A1 proteins using sodium dodecyl sulfate polyacrylamide gel electrophoresis (SDS-PAGE).

### Cell Sample Preparation and Enzymatic Activity Assay

A total of 150 µl of cOmplete Lysis-M (Merck, Darmstadt, Germany) was added to the pellet of HEK293, SH-SY5Y, and 1321N1 cells or human red blood cells. The mixture was sonicated, followed by centrifugation at 20,400 × *g* for 15 min. The supernatant was then collected from the cell lysate.

The activity of AKR1A1 was measured by monitoring NADPH consumption. The reaction mixture contained 100 mM HEPES (pH 7.4), 0.1 mM NADPH, and 10 mM GlucA. Reactions were monitored by assessing the decrease in absorbance at 340 nm using an Epoch Microplate Spectrophotometer (BioTek Instruments, Winooski, VT, United States) at 37°C. The enzyme activity was defined as the amount of enzyme that catalyzes the oxidation of 1 µmol of NADPH per min.

### RNA Extraction and Quantitative PCR

Total RNA was extracted from whole blood cell in using the NucleoSpin RNA Blood kit (NucleoSpin, MACHEREY-NAGEL, Duren, Germany) and the extracted RNA was purified using the RNeasy MinElute Cleanup Kit (QIAGEN, Hilden, Germany) according to the manufacturer’s protocol. The obtained RNA was quantified using NanoDrop spectrophotometer (Thermo Fisher Scientific, Waltham, MA, United States), and 500 ng of the purified RNA was used as a template for cDNA synthesis using ReverTra Ace qPCR RT Kit (Toyobo).

The qPCR assay was performed using Power SYBR Green PCR Master Mix (ThermoFisher Scientific). AKR1A1_qPCR_fw primer, AKR1A1_qPCR_rv primer, GAPDH_qPCR_fw primer and GAPDH_qPCR_rv primer were used (Supplementary Table S2). The qPCR protocol was as below: at 95°C for 10 min, followed by 40 cycles of 95°C for 15 s, 56°C for 30 s and 72°C for 30 s. For relative expression, each expression was normalized to average of two subjects without c.753G > A variant using the ΔΔC_T_ calculation.

### Statistical Analysis

The statistical tests were performed using GraphPad Prism version 9.0. Results are expressed as mean ± standard deviation (SD) or standard error of mean (SEM). Comparisons between groups were performed using two-way analysis of variance (ANOVA) or one-way ANOVA followed by Bonferroni’s multiple comparison test. Statistical significance was set at *p* < 0.05.

## Results

### Genetic Analysis of *AKR1A1*

**FIGURE 1:**
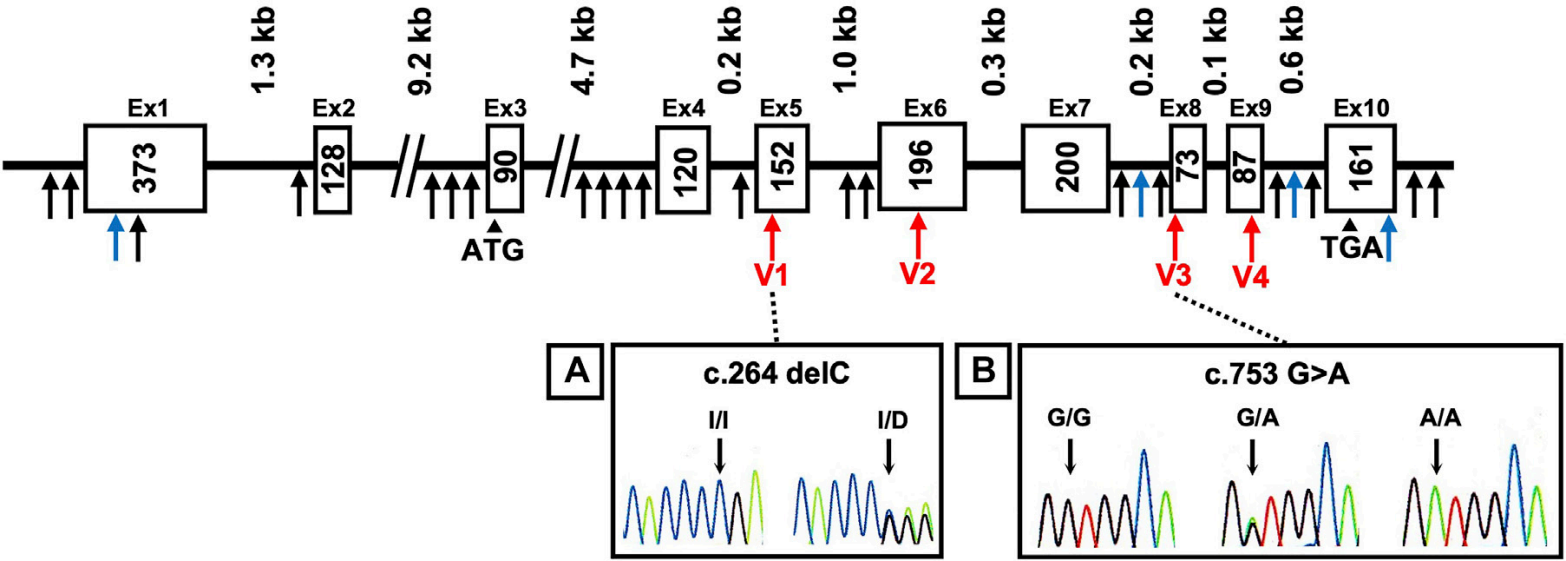
DNA sequence chromatograms showing frameshift mutation and variants. Novel variants (blue arrows), variants in coding regions shown in Table 1 (V1–V4 variants: red arrows), and others (black arrows) are shown. Heterozygous sequence traces derived from individuals carrying **(A)** a cytosine deletion within exon 5 (V1 variant) and **(B)** a mutation from guanine to adenine at the first position of exon 8 (V3 variant). Sequencing analyses revealed normal (denoted I/I or G/G) and mutant (denoted delC or G > A) sequences; kb indicates the kilobase pairs. WT, wild-type.

The *AKR1A1* sequence was analyzed in patients with schizophrenia (*n* = 808) and control subjects (*n* = 636), and 28 variants were identified as a result (Supplementary Table S3). Among them, four were novel variants: g.-403_-397 del AAGTTC, g.753-62_65 delC, g.913-114 C > A, and *85 G > C. The other variants have already been registered with the single nucleotide polymorphism database dbSNP (https://www.ncbi.nlm.nih.gov/snp/). In addition, four variants were found in the coding region: c.264 delC (rs755292778, p.Glu89ArgfsTer23), c.474 G > A (rs147059021, p.Ala158Ala), c.753 G > A (rs745484618, p.Arg251Arg), and c.911 C > T (rs150392728, p.Thr304Met), although the frequencies of the genotypes and alleles in the four variants did not noticeably differ between patients with schizophrenia and healthy controls (Table 1; Figure 1).

**TABLE 1:** Genotype and allele frequency of variants detected in *AKR1A1*.

	Nucleotide change, effect on protein (rs number)	gnomAD ID	Exon		N	Genotype counts (frequency)			*p* value	Effect size (Cramer’s V)	Allele counts (frequency)^b^		p value^c^	Effect size (Φ)
V1^a^	c.264 del C	1-45566924AC-A	Exon5			II	ID	DD			I	D		
				SCZ	758	757 (0.999)	1 (0.001)	0 (0)			1,515 (0.999)	1 (0.001)		
				CON	617	617 (1)	0 (0)	0 (0)	>0.999	0.024	1,234 (1)	0 (0)	> 0.999	0.017
				East Asian	2,588	2,587 (1)	1 (0)	0 (0)	0.402	0.016	5,175 (1)	1 (0)	0.402	0.011
				Total	76,027	76,026 (1)	1 (0)	0 (0)	0.020*	0.025	1,52,053 (1)	1 (0)	0.020*	0.018
V2	c.474 G>A Ala158Ala (rs147059021)	1-45568099G-A	Exon6			GG	GA	AA			G	A		
				SCZ	638	638 (1)	0 (0)	0 (0)			1,276 (1)	0 (0)		
				CON	549	548 (0.998)	1 (0.002)	0 (0)	0.464	0.031	1097 (0.999)	1 (0.001)	0.463	0.022
				East Asian	2,589	2,589 (1)	0 (0)	0 (0)	>0.999	0.000	5,178 (1)	0 (0)	> 0.999	0.000
				Total	76,047	75,999 (0.999)	48(0.001)	0 (0)	>0.999	0.002	1,52,046 (1)	48 (0)	> 0.999	0.002
V3	c.753 G>A Arg251Arg (rs745484618)	1-45568927G-A	Exon8			GG	GA	AA			G	A		
				SCZ	745	731 (0.981)	13 (0.017)	1 (0.001)			1,475 (0.99)	15 (0.01)		
				CON	617	612 (0.992)	5 (0.008)	0 (0)	0.191	0.048	1,229 (0.996)	5 (0.004)	0.074	0.035
				East Asian	2,600	2,591 (0.997)	9 (0.003)	0 (0)	<0.001***	0.079	5,191 (0.998)	9 (0.002)	<0.001***	0.058
				Total	76,076	76,067 (1)	9 (0)	0 (0)	<0.001***	0.107	1,52,143 (1)	9 (0)	<0.001***	0.078
V4	c.911 C > T Thr304Met (rs150392728)	1-45569228C-T	Exon9			CC	CT	TT			C	T		
				SCZ	745	743 (0.997)	2 (0.003)	0 (0)			1,488 (0.999)	2 (0.001)		
				CON	617	617 (1)	0 (0)	0 (0)	0.504	0.035	1,234 (1)	0 (0)	0.504	0.029
				East Asian	2,595	2,584 (0.996)	11 (0.004)	0 (0)	0.745	0.010	5,179 (0.998)	11 (0.002)	0.745	0.007
				Total	76,063	76,015 (0.999)	48 (0.001)	0 (0)	0.085	0.008	1,52,078 (1)	48 (0)	0.085	0.006

a The variants detected in the coding region are described using gene region, genotype counts, and allele counts.

b Allele frequencies of East Asian and Total groups (including African/African-American, Amish, Ashkenazi Jewish, East Asian, Finnish, non-Finnish European, Latino/Admixed American, Middle Eastern, Other, and South Asian) based on Genome Aggregation Database (gnomAD) were also used as controls. GRCh38 was used as a reference genome.

c The chi-squared test was used for statistical analysis when all cells had an expected value of more than 5. Fisher’s exact test was used when one or more cells had an expected value of 5 or less. *p < 0.05, ***p < 0.001 versus control.

The c.753G > A variant is a silent mutation. This variant was identified in 14 cases in the schizophrenia patient group (including 13 heterozygous and one homozygous) and five subjects in the healthy group, but no remarkable difference in genotype (*p* = 0.191) or allele frequency (*p* = 0.074) was observed. Next, we applied the frequency of the variants to “East Asian” and “Total groups (including African/African-American, Amish, Ashkenazi Jewish, East Asian, Finnish, non-Finnish European, Latino/Admixed American, Middle Eastern, Other, and South Asian)” obtained from Genome Aggregation Database (gnomAD) v3.1.1, a public database containing human genome data, as the frequency of control. The results showed that the frequency of genotypes GA and AA was significantly higher in patients than that in “East Asian” and “Total groups.” In addition, the frequency of allele A in patients was significantly higher than that in “East Asian” and “Total groups.”

The c.264delC variant was identified as a deletion-type mutation, which can have a significant impact on the expression product. This was observed in only one patient with schizophrenia in our sequence analysis. There was no significant difference in the genotype and allele frequencies between patients and controls in our dataset. However, further analysis using the database gnomAD showed that the frequency of genotype ID and allele D in patients was significantly higher than that in “Total groups,” but not “East Asian.”

The c.474G > A variant is a silent mutation without amino acid substitution. It was only found in one healthy person. In addition, the c.911C > T variant was identified in two patients in the schizophrenia patient group (heterozygous), and it was not identified in the healthy group. Regarding these variants, there was no significant difference in the frequency of the genotype and allele between patients and controls both in our dataset and gnomAD.

### Effect of the Mutation at the First Position of *AKR1A1* Exon on Exon Skipping and Decrease in Enzymatic Activity

**FIGURE 2:**
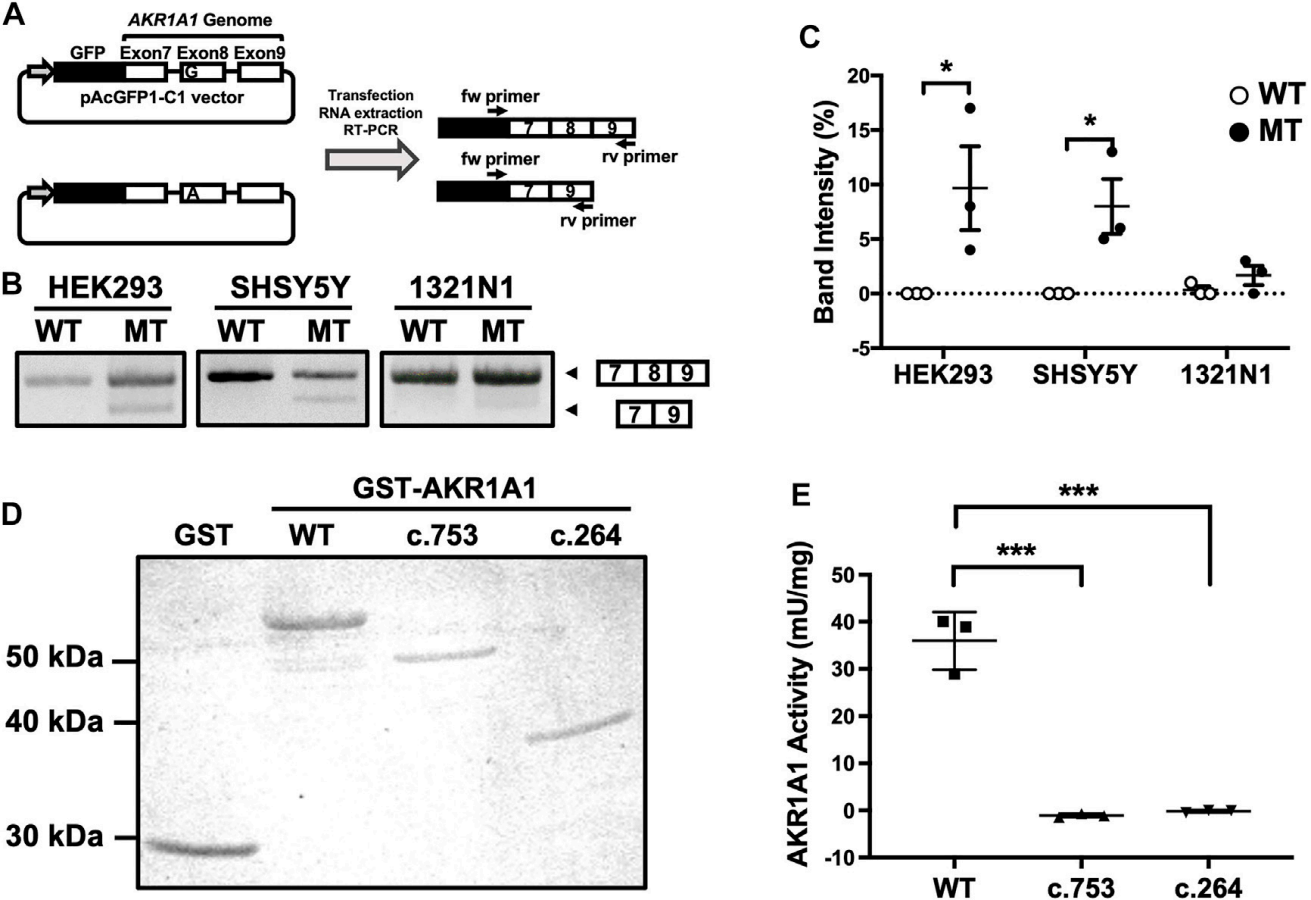
Exon skipping induced by the c.753G > A variant in *AKR1A1*. **(A)** An outline of the splicing assay is shown. Exon 8 skipping was confirmed using cDNA generated from HEK293, SH-SY5Y, and 1321N1 cells expressing minigenes for WT or c.753G > A variant (mutant [MT]). **(B)** The results of the splicing assay are shown. The upper band indicates products including exons 7–9, whereas the lower band shows exon 8 skipping products, including only exons 7 and 9. **(C)** Data represent the mean of three independent experiments for the splicing assay. Two-way analysis of variance: *F*_Interaction(2,12)_ = 2.65, *p >* 0.05; *F*_Cell(2,12)_ = 2.21, *p* > 0.05; *F*_Variant(1,12)_ = 16.4, *p* < 0.01. **(D)** GST and GST-AKR1A1s were purified and separated by sodium dodecyl sulfate polyacrylamide gel electrophoresis. **(E)** AKR1A1 activity of the purified GST-AKR1A1s was determined. Data represent the mean ± standard error of mean. *p < 0.05, ***p < 0.001 versus WT. AKR1A1, aldo-keto reductase family one member A1; WT, wild-type; GST, glutathione-S-transferase.

A mutation at the first position of an exon has been reported to cause exon skipping by alternative splicing (Fu et al., 2011). To address whether the variant c.753G > A located at the first position of exon 8 in the *AKR1A1* gene results in exon skipping, a minigene assay was performed. We constructed minigenes by inserting *AKR1A1* exons 7, 8, and 9 and the internal introns into the pAc-GFP1-C1 vector and by introducing the G > A mutation at the first position of exon 8 (Figure 2A). The minigenes were transfected into HEK293, SH-SY5Y, and 1321N1 cells, and their products were confirmed by PCR. The results showed that exon 8 skipping occurred in the A allele minigene (mutant [MT]) but not in the G allele (WT) in any cell type (Figure 2B). In HEK293 and SH-SY5Y cells, MT considerably increased the frequency of exon skipping compared to WT (Figure 2C). These findings suggest that the variant c.753G > A in the *AKR1A1* gene induced the skipping of exon 8.

The skipping of exon 8 causes a frameshift mutation and may result in the decreased enzymatic activity of AKR1A1 due to the c.753G > A variant, even though it is a silent mutation. To confirm this, we purified the AKR1A1 mutant recombinant protein produced by exon skipping and evaluated its activity (Figure 2D). We found that AKR1A1 produced by the c.753G > A variant exhibited no activity, which was significantly lower than that of the WT AKR1A1 (Figure 2E). Furthermore, c.264 delC mutation, which cause a frameshift mutation, also resulted in a complete loss of AKR1A1 enzymatic activity.

### Effect of the Mutation at the First Position of Exon in *AKR1A1* on Enzymatic Activity in Human Red Blood Cells

**FIGURE 3:**
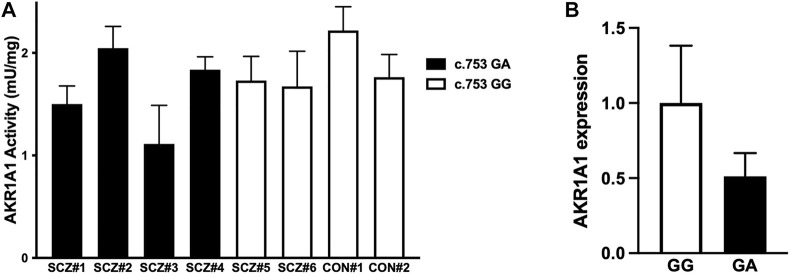
AKR enzymatic activity and *AKR1A1* gene expression in human. **(A)** The enzymatic activities of AKR in the red blood cells of six patients with schizophrenia (SCZ#1 to #4 with the c.753G > A variant and SCZ#5 and #6 without the variant) and two control subjects (CON#1 and #2 without the variant) were examined. Data represent mean ± SD of three independent experiments. **(B)** The *AKR1A1* mRNA expression in five subjects (SCZ#1, SCZ#3, SCZ#4, SCZ#6, and CON#2) was quantified by qPCR. For relative expression, each expression was normalized to average of SCZ#6 and CON#2 with GG alleles using the ΔΔC_T_ calculation. Data represent mean ± SD.

To investigate the effect of the c.753G > A variant on AKR1A1 enzymatic activity in patients with schizophrenia, AKR enzymatic activity in red blood cells from six patients with schizophrenia and two control subjects was measured (Figure 3A). Two out of four patients who were heterozygous at the c.753G > A variant exhibited lower enzymatic activity (patients with schizophrenia [SCZ]#1: 1.50 mU/mg protein, SCZ#3: 1.11 mU/mg protein), and a slight decrease was observed in patients with the variant compared to those without it (SCZ/GA: 1.62 ± 0.41 mU/mg protein; SCZ/GG: 1.70 ± 0.04 mU/mg protein). In addition, the enzymatic activity in four patients with GA was slightly lower than that in four subjects with GG (GA: 1.62 ± 0.41 mU/mg, GG: 1.85 ± 0.25 mU/mg). Furthermore, the enzymatic activity in patients with schizophrenia was slightly lower than that in control subjects (SCZ: 1.65 ± 0.32 mU/mg protein; control: 1.99 ± 0.32 mU/mg protein). Although there was no significant difference between any of the groups due to the small number of subjects, these results suggest that the c.753G > A variant of *AKR1A1* may reduce the enzymatic activity of AKR1A1.

Finally, to investigate the effect of the c.753G > A variant on *AKR1A1* expression, we quantified mRNA level in the whole blood cells obtained from five subjects (CON#2 and SCZ#6 with c.753 GG alleles, and SCZ#1, SCZ#2, and SCZ#4 with c.753 GA alleles), since unfortunately we were unable to collect peripheral blood from three individuals whose AKR activity we have measured. We found that mRNA expression of *AKR1A1* with c.753 GA alleles decreased to approximately 50% compared to that with c.753 GG (Figure 3B). These results suggest that the reduction of AKR activity in subjects with the variant may be caused from the reduction of gene expression.

## Discussion

In the present study, we identified 28 variants of the *AKR1A1* (Figure 1; Supplementary Table S1). Among them, we found that two variants in the coding region were significantly associated with schizophrenia using the database gnomAD: the frameshift mutation in exon 5 (c.264delC, p.Glu89ArgfsTer23) and the exon-skipping mutation in exon 8 (c.753 G > A, p.Arg251Arg). The c.264delC allele frequency in patients with schizophrenia was significantly higher than that in healthy controls in “Total groups” populations in the database gnomAD (Table 1; *p* = 0.020). According to the Japanese multi omics reference panel including 8380 healthy subjects (jMorp: https://jmorp.megabank.tohoku.ac.jp.), the c.264delC allele frequency in Japanese is 0.0001. As the c.264delC variant produces a termination codon in the middle of the gene due to a frameshift, its aberrant product may undergo degradation via a nonsense-mediated mRNA decay (NMD) mechanism (Kishor et al., 2019). In one heterozygous case with c.264delC, a decrease in expression and enzymatic activity of the AKR1A1 protein and the subsequent GlucA accumulation was expected, but it could not be confirmed because of difficulty in re-recruiting the subject.

The c.753 A allele frequency in patients with schizophrenia was significantly higher than that in healthy controls in “East Asian” and “Total groups” populations in the database gnomAD (Table 1; *p* < 0.001, *p* < 0.001, respectively). According to the jMorp, the c.753A allele frequency in Japanese was 0.0037, which means that it is relatively common among Japanese people compared to the world. Furthermore, three patients from the same family (mother and two identical sons) were found to possess the c.753G > A variant. These findings suggest that the c.753G > A variant may be one of the genetic factors related to schizophrenia.

The g.-275 T allele frequency in patients with schizophrenia was significantly higher than that in healthy controls in “East Asian” and “Total groups” populations in the database gnomAD (Supplementary Table S3; *p* = 0.020 and *p* < 0.001, respectively). It is located in untranslated region, but it has been reported that this variant is associated with negative symptoms of schizophrenia (Martinez-Magana et al., 2021).

It should be noted that the c.753G > A mutation is located at the first position of exon 8, although the variant itself is a silent mutation. We revealed that this variant led to exon 8 skipping using a splicing assay with minigenes (Figures 2A–C). In the splicing process, splicing factors U2AF^35^ and U2AF^65^ bind to the 5′ exon (3′ splice) site and the polypyrimidine tract in the intron, respectively (Warnasooriya et al., 2020). When the polypyrimidine tract is sufficiently long (10–15 nt), U2AF^65^ binds strongly and normal splicing occurs even without U2AF^35^ binding. However, in cases where the polypyrimidine chain is short, the binding of U2AF^35^ to the 5′ exon site is required for splicing. If the polypyrimidine chain is short and there is a mutation in the first position of the exon, both U2AF^65^ and U2AF^35^ cannot bind to the splicing site, resulting in exon skipping (Fu et al., 2011). Therefore, the exon-skipping frequency depends on the length of the polypyrimidine tract and the 5′ exon site. Our findings showing the c.753G > A mutation at the first position of exon 8 are consistent with the splicing mechanism because the polypyrimidine tract upstream of exon 8 in *AKR1A1* is as short as 6 nt. Furthermore, the c.753G > A variant induced exon skipping in HEK293 and SH-SY5Y cells, but not in 1321N cells (Figures 2B,C). This difference in the frequency of exon skipping among these cell types may be due to differences in the expression of splicing factors such as U2AF^35^ and U2AF^65^.

Exon 8 skipping in *AKR1A1* caused a frameshift mutation, resulting in a truncated AKR1A1 protein. The normal protein of AKR1A1 consists of 325 amino acids, but when the c.753G > A variant causes a frameshift, the first 88 amino acids from the N-terminus are the same as those in the normal protein, but the rest are lost, resulting in a truncated protein with ten extra amino acids (total of 261 amino acids). As for the c.264delC variant, the first 88 amino acids are the same as those in the normal protein, but the rest are lost, resulting in a truncated protein with 22 extra amino acids (total of 110 amino acids). Using the recombinant protein, we demonstrated that the truncated AKR1A1 proteins produced by c.753G > A and c.264delC variants completely lost their enzymatic activity (Figures 2D,E). These findings suggest that patients with c.753G > A and c.264delC variants in *AKR1A1* might show GlucA accumulation in the peripheral blood. Moreover, it is known that AKR1A1 is involved in the metabolism of the neurotransmitter noradrenaline and the carbonyl compound methylglyoxal (Sato et al., 1999; Vander Jagt et al., 1992). Noradrenaline levels are reduced in patients with schizophrenia, and negative symptoms are improved by inhibiting noradrenaline reuptake (Matthews et al., 2018; Catak et al., 2019). Therefore, the frameshift mutations in AKR1A1 might affect these metabolic processes, and thus may be related to the pathophysiology of schizophrenia.

Furthermore, we found that AKR activity tended to be lower in patients with the c.753G > A mutation than that in healthy controls (Figure 3A). Furthermore, *AKR1A1* mRNA expression in the whole blood cells of individuals with the c.753G > A variant tended to be lower than that in those without the variants, leading to lower AKR activity (Figure 3B). This is consistent with the findings of our *in vitro* analyses (Figure 2). These findings suggest that the aberrant product caused by c.753 G > A variants may undergo degradation probably via NMD, leading to decreased AKR activities. However, as seen in Figure 2C, the occurrence of exon skipping due to the allele A is about 10–20% at most, which is not very frequent. The result suggests the reduced expression of *AKR1A1* in patients with the c.753G > A variant is not entirely due to the variant. Perhaps an unknown molecular pathology present in schizophrenia patients is involved in the decreased expression of *AKR1A1*. Further studies with larger populations will be needed in the future to clarify this point.

A detailed clinical information of three (SCZ#1, SCZ#3 and SCZ#4) out of four patients harboring mutant type allele at c.753G > A revealed that all the patients exhibited treatment resistant (TR) phenotype. Of these patients, SCZ#1 and SCZ#3 showed marked and moderate reduction of enzymatic activity of AKR1A1 compared to that of patients with wild type allele, respectively. In particular, maintenance electroconvulsive therapy has been necessary for SCZ#3 to prevent the relapse because any therapeutic agents have no efficacy for the psychotic symptoms. Therefore, elevated GlucA may contribute to the pathophysiology of TR phenotype for these patients due to diminished enzymatic activity of AKR1A1 caused by mutation at c.753G > A. In contrast to these patients, AKR1A1 activity of SCZ#4 was comparable with that of patients with wild type. Thus, underlying mechanism of TR phenotype for SCZ#4 may be different from that of SCZ#3 and SCZ#1.

The enzymatic activity of patient SCZ#2 is not reduced, even though the patient has the variant (Figure 3A). However, due to the lack of detailed clinical information and difficulty in re-recruiting the patient SCZ#2, we were unable to conduct further analysis, including *AKR1A1* expression analysis. It is one of the limitations of this study, and we believe that the unaltered enzymatic activity of SCZ#2 may be due to epigenetic effects or other cofactors.

In addition, this study data has other notable limitations. First, the number of patients and healthy subjects whose AKR activity could be measured was extremely small. Second, we cannot measure the GlucA level in the blood of patients with the c.753G > A mutation, so we have not been able to evaluate the actual effect of the mutation on glucuronic acid accumulation. In the future, a higher number of patients must be considered for further investigation and the link between the frameshift mutations in AKR1A1 and the aforementioned metabolic processes and whether GlucA accumulation occurs in patients with these variants should be clarified.

Among the identified variants of *AKR1A1* related to schizophrenia, the c.753G > A variant caused exon skipping, leading to the production of a truncated AKR1A1 protein. We found that this truncated AKR1A1 protein lost its enzymatic activity, probably leading to the accumulation of GlucA. Notably, we revealed that AKR activity tends to be reduced in patients with the c.753G > A mutation compared with healthy controls, suggesting that patients with schizophrenia with the c.753G > A variant might show higher GlucA levels, leading to drug-resistant schizophrenia, since drug excretion by GlucA is enhanced.

## Data Availability

The datasets presented in this study can be found in online repositories. The names of the repository/repositories and accession number(s) can be found in the article/Supplementary Material.
